# Supplementary material for: Cloud BioLinux: pre-configured and on-demand bioinformatics computing for the genomics community
Source: BMC Bioinformatics. 2012 Mar 19;13:42. doi: 10.1186/1471-2105-13-42 (PMC3372431; doi:10.1186/1471-2105-13-42)
Supplement: Additional file 1 — Supplementary 1 Cloud BioLinux software documentation in the form of a mini, self-contained website. Users need to download and uncompress the .zip file, and open through a web browser the "index.html" file available on the main directory. (ZIP 1823 kb). [file 1471-2105-13-42-S1.ZIP › Cloud-BioLinux-Package-Documentation/docs/phrap.html]

Bio-Linux Software Documentation Pages

Back to search form

## phrap

|  |  |
| --- | --- |
| Name | phrap |
| Description | **Phrap** is a program for assembling shotgun DNA sequence data. It allows use of entire sequence reads (not just the trimmed high quality part), it uses a combination of user-supplied and internally computed data quality information to improve assembly accuracy in the presence of repeats, it constructs the contig sequence as a mosaic of the highest quality read segments rather than a consensus, it provides extensive assembly information to assist in trouble-shooting assembly problems, and it handles large datasets.  **Phrap** is required and/or recommended by some packages available on Bio-Linux such as gap4 (Staden), stars, and partigene. Please also see the documentation on **gcphrap** if you use the Staden package.  **Phrap** is ***licensed software*** and thus is ***not*** installed by default on Bio-Linux. It is free to academic researchers but you must obtain a license. Further information on how to obtain phrap is available on the official website for phred, phrap and consed.  If and only if you have already obtained a license to use phred or phrap (cross\_match is found within the phrap package), the NEBC can provide you with packages that will install these programs onto Bio-Linux, including the staden-specific phrap extra: gcphrap. Please contact the NEBC helpdesk if you have your license and are ready to proceed. |
| Homepage | http://www.phrap.org |
| Remote Documentation | http://www.phrap.org/phredphrapconsed.html#block\_phrap |
